# Thermal and Chemical Stability of Two Homologous POZ/BTB Domains of KCTD Proteins Characterized by a Different Oligomeric Organization

**DOI:** 10.1155/2013/162674

**Published:** 2013-11-06

**Authors:** Luciano Pirone, Carla Esposito, Stefania Correale, Giuseppe Graziano, Sonia Di Gaetano, Luigi Vitagliano, Emilia Pedone

**Affiliations:** ^1^Istituto di Cristallografia, CNR, Via G. Amendola 122/O, 70126 Bari, Italy; ^2^DFM Scarl, Via Mezzocannone 16, 80134 Napoli, Italy; ^3^Istituto di Biostrutture e Bioimmagini, CNR, Via Mezzocannone 16, 80134 Napoli, Italy; ^4^Kedrion S.p.A, S. Antimo, 80029 Napoli, Italy; ^5^Dipartimento di Scienze per la Biologia, la Geologia e l'Ambiente, Università del Sannio, Via Port'Arsa 11, 82100 Benevento, Italy

## Abstract

POZ/BTB domains are widespread modules detected in a variety of different biological contexts. Here, we report a biophysical characterization of the POZ/BTB of KCTD6, a protein that is involved in the turnover of the muscle small ankyrin-1 isoform 5 and, in combination with KCTD11, in the ubiquitination and degradation of HDAC1. The analyses show that the domain is a tetramer made up by subunits with the expected **α**/*β* structure. A detailed investigation of its stability, carried out in comparison with the homologous pentameric POZ/BTB domain isolated from KCTD5, highlights a number of interesting features, which are shared by the two domains despite their different organization. Their thermal/chemical denaturation curves are characterized by a single and sharp inflection point, suggesting that the denaturation of the two domains is a cooperative two-state process. Furthermore, both domains present a significant content of secondary structure in their denatured state and a reversible denaturation process. We suggest that the ability of these domains to fold and unfold reversibly, a property that is somewhat unexpected for these oligomeric assemblies, may have important implications for their biological function. Indeed, these properties likely favor the formation of heteromeric associations that may be essential for the intricate regulation of the processes in which these proteins are involved.

## 1. Introduction

A large fraction of proteins are oligomeric in their functional state(s). Although protein oligomerization may be incidental, several studies have highlighted the benefits of this process in a variety of different systems. In this framework, the analysis of the structural determinants of protein oligomerization is a field of considerable interest. The characterization of homologous proteins endowed with different quaternary structures is particularly suited to unveil key factors in protein oligomerization [1, 2].

Structural and functional literature studies have highlighted that specific domains, often shared by proteins involved in completely different biological processes, have been specifically designed by evolution to mediate protein-protein interactions and to promote oligomerization. One of the best characterized domains deputed to these roles is the POZ/BTB domain, a motif that is widespread among eukaryotes [3]. From the structural point of view, this domain has a well-defined tridimensional fold characterized by a large interaction interface, that facilitates intermolecular interactions. This external surface is highly modifiable through amino-acid substitutions; indeed, variations on this common theme lead to a variegate ensemble of either transient or firm protein associations. 

Among the proteins embedding POZ/BTB domain(s), the family denoted as KCTD, containing the potassium channel tetramerization domain, has recently received a special attention. Although the name of the family derives from their homology with proteins involved in the formation of voltage-gated K^+^ (Kv) channels, the twenty-three KCTD genes present in the human genome encode for proteins implicated in a variety of biological processes not connected with voltage channels. Recent investigations have shown that some members of the family play a key role in protein ubiquitination and degradation as they act as cullin binding adaptors (KCTD11, KCTD21, KCTD6, KCTD13, and KCTD10) [4–7]. In other members of the family, such as KCTD5, KCTD1, and KCTD7, that are known to bind cullin 3, are likely involved in similar processes [8–10]. Finally, some KCTDs (KCTD8, KCTD12, KCTD12b, and KCTD16) are integral constituents of native GABAB receptors [11]. KCTDs are involved in the insurgence and the progression of severe human pathologies including cancers, epilepsy and obesity [12–14]. Despite their biological relevance, the molecular and structural characterization of these proteins is still limited.

Structural information have been so far reported only for KCTD5, KCTD11 and KCTD12 [6, 15–17]. KCTD5, the sole member of the family whose three-dimensional structure has been experimentally determined, presents a pentameric association of both the POZ/BTB N-terminal and the C-terminal domains [16]. A biophysical characterization of KCTD11 has shown that the protein and its N-terminal POZ/BTB domain are tetrameric. This observation is rather surprising considering the high sequence identity of KCTD11 and KCTD5 POZ/BTB (KCTD5_BTB_) domains and that evolutionary transitions from tetrameric (point symmetry C4) to pentameric (point symmetry C5) associations are very unlikely [1]. In order to gain further insights into the puzzling organization of POZ/BTB domains of KCTD proteins, we report the biophysical characterization of the POZ/BTB of KCTD6 (KCTD6_BTB_), a member of the family that shares significant sequence and functional similarities with the well-characterized KCTD11. KCTD6 is a recently discovered substrate adaptor for cullin-3 that regulates protein levels of the muscle small ankyrin-1 isoform 5 (sAnk1.5) [18] and that, in combination with KCTD11, is also involved in ubiquitination and degradation of HDAC1, which is involved in the regulation of the acetylation state of the transcription factors Gli1 and Gli2 [4, 5, 19]. We then compared the thermal and the chemical stability of this tetrameric protein with the pentameric homolog POZ/BTB of KCTD5. Interestingly, both proteins exhibit reversible denaturation despite their oligomeric organization.

## 2. Materials and Methods

### 2.1. Cloning, Expression and Purification of KCTD6_BTB_ and KCTD5_BTB_

KCTD6_BTB_ (residues 10–110 of KCTD6) was amplified by PCR, using as template the human* KCTD6 *cDNA (NM_153331) (thermo Scientific) and cloned into the pETM-11 expression vector (Novagen). *Escherichia coli* BL21(DE3) Star strain (Invitrogen) was transformed with the recombinant vector, grown at 37°C and induced by 0.5 mM isopropyl-*β*-d-thiogalactoside (IPTG) for 16 h at 22°C. 

The cell pellets were re-suspended in 50 mM Tris-HCl, 0.1 M NaCl, pH 8.0. The KCTD6_BTB_ domain was purified as already described for KCTD11 with slight modifications [6].

The pET28/KCTD5_BTB_ plasmid was a gift of Prof. Goldstein (University of Chicago). The recombinant protein was expressed and purified according to the procedure previously reported. 

### 2.2. Static Light Scattering

Static light scattering experiments were performed using a MiniDAWN Treos spectrometer (Wyatt Instrument Technology Corp.) equipped with a laser operating at 658 nm and connected online to a size-exclusion chromatography.

Purified KCTD6_BTB_ was analyzed by size-exclusion chromatography connected to a triple-angle light scattering detector equipped with a QELS module (quasielastic light scattering) for mass value. 500 *μ*L sample (1 mg/mL) was loaded on a Superdex 75 10/30 column, equilibrated in 50 mM Tris-HCl and 0.15 M NaCl, pH 8.0 and analyzed as described elsewhere [6].

### 2.3. Spectroscopic Studies

CD spectra were recorded at 20°C using a Jasco J-810 spectropolarimeter equipped with a Peltier thermostatic cell holder. Far-UV measurements (260–190 nm) were carried out using a 0.1 cm path length cell in a 10 mM Tris-HCl, pH 8.0 buffer at a concentration of 5 *μ*M. 

Chemical denaturation was induced by guanidine hydrochloride (GuHCl) or urea in a range of 0 to 6.0 M and 0 to 8.0 M, respectively, incubating the samples 2 h at room temperature. All the denaturations were investigated by recording the CD signal at 222 nm.

Fluorescence experiments were conducted in the same conditions. Fluorescence spectra were collected at 20°C using a Varian Cary Eclipse spectrophotometer and a 1.0 cm path length cell. For KCTD5_BTB_, two separate sets of experiments were carried out setting the excitation wavelength at either 280 or 295 nm. For KCTD6_BTB_, that does not contain Trp residues, only the experiment with excitation at 280 nm was performed. In all cases, the emission was recorded in the range 290–450 nm.

## 3. Results

### 3.1. Sequence Analysis and Secondary Structure Content of KCTD6

KCTD6 is a protein of 237 residues (UniProt code Q8NC69). Comparative analyses of its sequence with those reported in the sequence databanks clearly indicate that the POZ/BTB domain, located in the N-terminal region of the protein, namely KCTD6_BTB_, spans from residue 10 to residue 110. Pair-wise alignments of KCTD6_BTB_ with POZ/BTB domains of other KCTDs unveil that the closest ones are those of KCTD11 and KCTD21. The sequence identity shared by KCTD11_BTB_ and KCTD21_BTB_ with KCTD6_BTB_ is 61% and 72%, respectively. KCTD6_BTB_ presents sequence identities higher than 50% when compared with KCTD1 (54%), KCTD4 (53%), and KCTD15 (57%).

To gain insights into the structural features of the protein, we performed a secondary structure prediction session on KCTD6 sequence by using the Swiss Model server (http://swissmodel.expasy.org/). The server predicts the occurrence of several *β*-strands and *α*-helices in KCTD structure (Figure S1) (Supplementary Material available online at: http://dx.doi.org/10.1155/2013/162674). The inspection of the region 10–110, corresponding to the BTB domain, unveils the presence of the structural elements (three *β*-strands and five *α*-helices) characteristic of this motif. The prediction also suggests that the C-terminal domain of the protein is highly structured, being characterized by the combination of *α*- and *β*-structure with coil regions of limited size.

### 3.2. Expression and Characterization of KCTD6_BTB_ Oligomerization State

**Figure 1:**
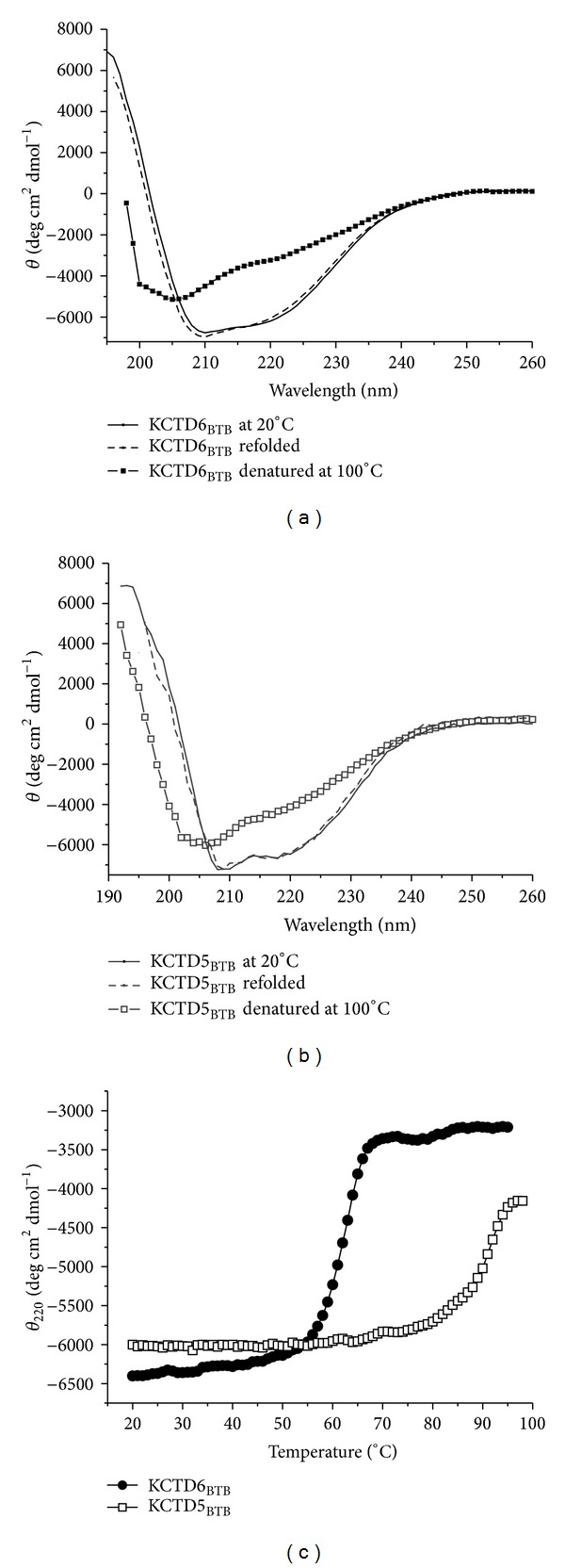
CD spectra of KCTD6_BTB_ (a) and KCTD5_BTB_ (b) curves recorded at 20°C, at 100°C, and after decreasing the temperature back to 20°C are indicated with solid, dash, and dash-dot lines, respectively. Thermal denaturation curves are shown in (c).

In order to perform a biophysical characterization of KCTD6_BTB_, this domain was cloned and expressed in *E. coli*. The particularly high yield (∼1 g/L) of the purified recombinant product allowed its extensive biophysical characterization. The homogeneity of the purified protein was assessed with a variety of different techniques (SDS PAGE and mass spectrometry). The far-UV CD spectrum of KCTD6_BTB_ is consistent with a properly folded protein (Figure 1(a)). The spectrum is characterized by the presence of two minima (at 210 and 219 nm) and one maximum (at 195 nm) which are typical fingerprints of *α*/*β* proteins.

**Figure 2:**
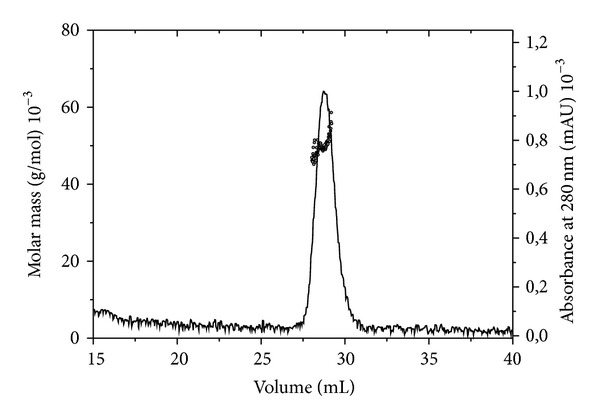
Static light scattering analysis (molar mass versus elution volume) of KCTD6_BTB_.

As POZ/BTB domains of KCTD proteins may be characterized, despite their high sequence identities, by different oligomeric states, we carefully investigated the aggregation state of KCTD6_BTB_. Light scattering measurements provided a weight-average molar mass for KCTD6_BTB_ of 50580 ± 350 Da (Figure 2). These experiments suggest a tetrameric organization of the protein. This finding is in line with gel filtration experiments (Table S1). These results are not surprising as KCTD6_BTB_ shares a very high sequence identity with KCTD11_BTB_ (61%) and KCTD21_BTB_ (72%),which are also tetrameric in solution [6]. It is also worth mentioning that the residues that stabilize the pentameric interface in KCTD5 are not conserved in KCTD6.

### 3.3. Thermal Denaturation: KCTD6_BTB_ versus KCTD5_BTB_

Thermal denaturation analyses of the KCTD6_BTB_ domain were conducted by using far-UV CD spectroscopy by following the CD signal at 222 nm. As the tetrameric KCTD6_BTB_ shares a significant sequence identity (approx. 40%) with the pentameric KCTD5_BTB_  (Figure S2), the analyses were extended to the latter domain for comparative purposes. The far-UV CD spectrum of the KCTD6_BTB_ sample kept at 100°C is different from that recorded for the native form at 20°C, but is characterized by the presence of a broad minimum at 205 nm and a shoulder at 220 nm (Figure 1(a)). The latter features are indicative of the persistence of secondary structure elements in the thermally denatured form of the domain. Interestingly, when the temperature is decreased back to 20°C, KCTD6_BTB_ fully recovers its initial structure, as indicated by the far-UV CD spectrum (Figure 1(a)). This demonstrates that the thermal denaturation of KCTD6_BTB_ is a reversible process. As shown in Figure 1(c), the thermal denaturation curve of KCTD6_BTB_ presents a sigmoidal shape with a single inflection point, corresponding to a melting temperature (*T*_*m*_) of 62°C, suggestive of a cooperative process in which dissociation and unfolding are coupled. The latter mechanism is supported by the fact that, on increasing the protein concentration, the melting curves present the same shape with an increase in the melting temperature (data not shown), as it should be for a reversible two-state process in which dissociation and unfolding are coupled [20, 21].

Similar analyses conducted on KCTD5_BTB_ unveil differences and analogies when compared to KCTD6_BTB_. The overall shape of the far-UV CD spectrum of KCTD5_BTB_ is similar to that of KCTD6_BTB_ (Figure 1(b) and Figure S3) suggesting an analogous secondary structure content, in line with the high sequence identity. As in the case of KCTD6_BTB_: (a) the thermally-denatured state of KCTD5_BTB_ is characterized by the presence of a significant content of residual secondary structure and (b) the KCTD5_BTB_ thermal denaturation is also reversible, since the secondary structure is recovered when temperature is lowered to 20°C (Figure 1(b)). As shown in Figure 1(c), the thermal denaturation curve of KCTD5_BTB_ also presents a sigmoidal shape with a single inflection point, suggestive of a cooperative process in which dissociation and unfolding are coupled. However, CD measurements show that the pentameric KCTD5_BTB_ is significantly more stable than the tetrameric KCTD6_BTB_. Indeed, when the stability of KCTD5_BTB_ is measured using the same concentration used for KCTD6_BTB_, its *T*_*m*_ value is as high as 93°C (Figure 1(c)). Therefore, reversibility and cooperativity of the temperature-induced denaturation (i.e., a reversible two-state process in which dissociation and unfolding are coupled) is a common feature of KCTD5_BTB_ and KCTD6_BTB_, despite their complex and diversified oligomeric organization.

### 3.4. Chemical Denaturation: KCTD6_BTB_ versus KCTD5_BTB_

**Figure 3:**
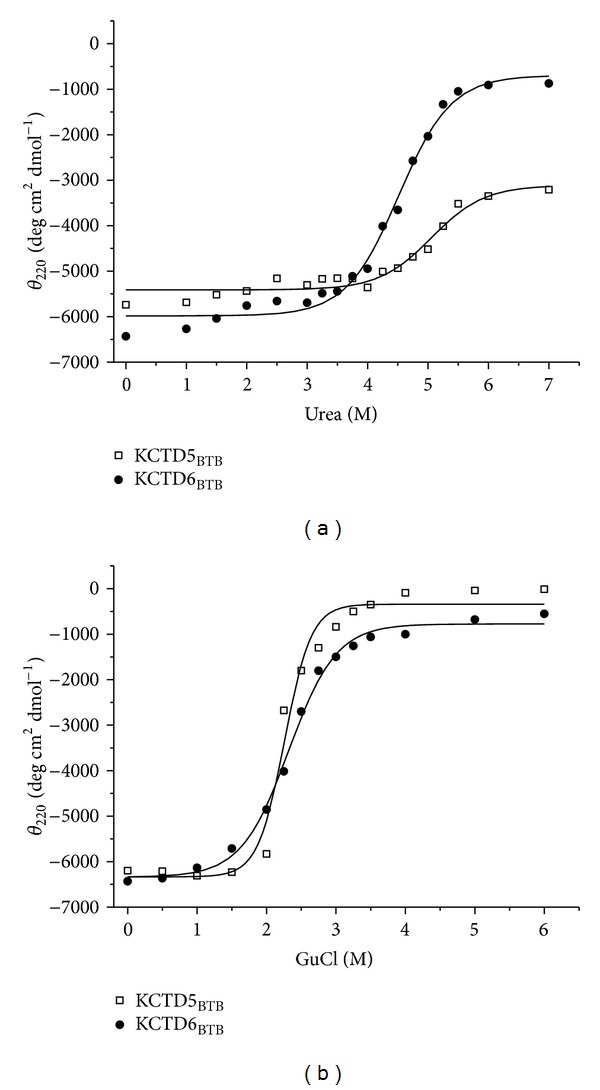
Chemical denaturation induced by urea (a) or GuHCl (b) of KCTD6_BTB_ and KCTD5_BTB_ followed by CD spectroscopy.

The comparative analysis of the conformational stability of KCTD6_BTB_ and KCTD5_BTB_ was extended by performing chemical denaturation experiments using either urea or GuHCl as denaturant and the same protein concentration. To gain insights into these events at the level of both secondary and tertiary structure, the conformational stability of the two domains against the denaturing action of urea and GuHCl was investigated by recording (a) the change in molar ellipticity at 222 nm and (b) the change in fluorescence spectrum upon excitation at either 280 nm or 295 nm. The evolution of the CD signal at 222 nm upon the addition of the denaturants is characterized by a sigmoidal shape, with a single inflection point (Figure 3), and the denaturation proves to be reversible for both proteins against both denaturants. The transition is sharper upon the addition of GuHCl, although the process does appear to be rather cooperative for both denaturants. Despite the differences exhibited in the thermal denaturation analysis, the two domains are characterized by a roughly similar stability against the denaturing action of urea and GuHCl, respectively. Indeed, the C_1/2_ value exhibited by the two domains upon addition of urea is 4.7 M for KCTD6_BTB_ and 5.0 M for KCTD5_BTB_. As expected, the C_1/2_ value, which is 2.2 M for both proteins, is lower in the presence of GuHCl. The non-zero CD signal at 222 nm, observed at very high urea concentration, suggests that the urea-denatured state is characterized by a residual secondary structure content. This is particularly evident in the case of KCTD5_BTB_. When GuHCl is used as denaturant, the content of residual secondary structure is marginal for both domains.

**Figure 4:**
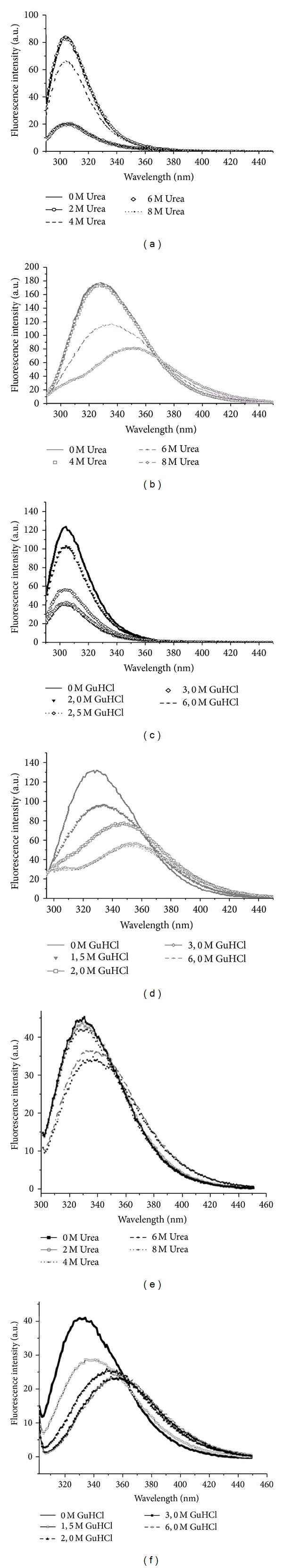
Chemical denaturation of KCTD6_BTB_ and KCTD5_BTB_ followed by fluorescence. The spectra obtained upon excitation at 280 nm in the presence of increasing concentration of urea are reported in (a) and (b) for KCTD6_BTB_ and KCTD5_BTB_, respectively. Similarly, the spectra obtained upon excitation at 280 nm in the presence of increasing concentration of GuHCl are reported in (c) and (d), for KCTD6_BTB_ and KCTD5_BTB_, respectively. Spectra obtained for KCTD5_BTB_ after excitation at 295 nm are reported in (e) (urea) and (f) (GuHCl).

The intensity of the fluorescence spectra of the two domains shows clear variations when the concentration of the denaturant approaches the C_1/2_ values determined in the CD analysis (Figure 4). This observation strongly suggests that structural transitions at the secondary and tertiary level are concomitant and lead to a cooperative and reversible process. A comparative analysis of the fluorescence spectra of the two domains shows some significant differences. The variation of the intensity in the fluorescence spectra of KCTD6_BTB_, which were recorded exclusively upon excitation at 280 nm is not coupled with changes in the wavelength of the maximum that remains close to 305 nm. On the other hand, KCTD5_BTB_ fluorescence spectra collected in similar conditions are characterized by a red shift of the wavelength of the maximum, while the intensity decreases. Since KCTD5_BTB_ sequence contains a single Trp residue (Trp45), fluorescence spectra were also collected upon excitation at 295 nm. These spectra show trends similar to those recorded upon excitation at 280 nm. The addition of both denaturants produces a decrease of the intensity and a shift of the wavelength of the maximum from 330 to 360 nm. This finding indicates that the side chains of the five Trp present in KCTD5_BTB_ pentamer, that are buried in the native structure, become solvent exposed in the denatured state. Since Trp45 is located in the initial strand of the sole *β*-sheet present in KCTD5_BTB_ structure, this finding may suggest that the *β*-structure is highly perturbed in the denaturation and that the residual structure present in the denatured state corresponds to helical regions. It is also likely that the differences observed in the fluorescence spectra of the two proteins, recorded upon excitation at 280 nm, may be due to the contribution of the Trp45 side chains in the KCTD5_BTB_ domain.

## 4. Discussion

POZ/BTB domains are versatile modules that are often implicated in fundamental biological processes [22]. Previous literature data have shown that these domains are able to self-associate and to form specific homo- and hetero-oligomers [22]. In this framework, the elucidation of the biophysical properties of these domains represents an important step for a full understanding of their role and for designing *ad hoc* strategies aimed at modulating their activities. Here, we report a characterization of two POZ/BTB domains (KCTD6_BTB_ and KCTD5_BTB_) isolated from proteins belonging to the family of KCTDs, an emerging class of key factors involved in severe human pathologies. Although KCTD6_BTB_ and KCTD5_BTB_ share a high sequence identity (>40%), these domains are characterized by a different oligomeric organization. Indeed, literature data have shown that KCTD5_BTB_ is pentameric [6, 16], whereas present data indicate that KCTD6_BTB_ is tetrameric. The tetrameric association of KCTD6_BTB_ is not surprising taking into account the high sequence identity (>61%) that this domain shares with the tetrameric POZ/BTB domains of KCTD11 and KCTD21. Moreover, the analysis of KCTD6_BTB_ sequence indicates that this domain does not contain the residues that are believed to stabilize the pentameric association of KCTD5_BTB_(Figure S2) [16]. The finding that these two domains adopt a different oligomeric organization, despite the high sequence homology, is even more puzzling considering that they play a similar biological function: the recruitment of cullin 3 in the E3 ligase complex [4, 18]. It is important to note that from the evolutionary point of view, the transition in homologous proteins from cyclic tetrameric to cyclic pentameric states is a very rare (low probability) event [1].

Even though both domains display a remarkable stability against temperature, a significant difference is emerged: the melting temperature is 62°C for KCTD6_BTB_ but 93°C for KCTD5_BTB_. Although the absence of structural data for KCTD6_BTB_ hampers the possibility to relate this marked difference to specific structural determinants, it is likely that the peculiar stability of KCTD5_BTB_ may be related, among other factors, to the presence of an intrasubunit disulfide bridge that connects Cys62 to Cys74 [16]. In addition, the analysis of the 3D structure shows that each subunit is involved in extensive interactions in the pentameric organization. Indeed, approximately 25% of the surface of each subunit (about 1500 Å^2^ out of 6000 Å^2^) is buried upon pentamer formation.

On the other hand, it is important to note that the stability of KCTD5_BTB_ against chemical denaturants (urea and GuHCl) is less remarkable. KCTD6_BTB_ and KCTD5_BTB_ show similar C_1/2_ values, close to 5.0 M and 2.2 M, when they are denatured by means of urea and GuHCl, respectively. The relatively low value of the ratio C_1/2_(urea)/C_1/2_(GuHCl) ≈ 2.2 suggests that electrostatic interactions, that are efficiently impaired by GuHCl, should play a limited role in the stabilization of these oligomeric structures [23, 24]. The analysis of the thermal/chemical denaturation of these domains also highlights a number of intriguing properties, which are shared by the two domains despite their different oligomeric organization. Present data indicate that (a) thermal/chemical denaturation is a reversible process for both domains (b) thermal/chemical denaturation curves of both proteins are characterized by a single and sharp inflection point, suggestive of a cooperative two-state process, in which dissociation and unfolding are coupled; and (c) both domains maintain a significant content of secondary structure in the thermally denatured state. It is likely that the presence of a residual structure in their denatured state could favor the recovery of the native structure in the refolding process.

The ability of these domains to fold and unfold in a reversible manner is somewhat unexpected for these oligomeric assemblies. Among the few literature examples, it is worth mentioning the tetramerization domain of p53 tumor suppressor protein which shows a reversible and cooperative two-state denaturation against both temperature and GuHCl [20, 25]. This property shared by both KCTD6_BTB_ and KCTD5_BTB_ may have important implications for their biological function. Recent literature data have shown that the POZ/BTB domains are used by KCTDs proteins to form heteromeric associations among different members of the family [4]. It has been shown, for example, that KCTD10 interacts with KCTD13 [26] and that KCTD6 is able to form heteromeric assemblies, likely tetramers, with KCTD11 and KCTD21 [4]. Since C-terminal domains of KCTD6 and KCTD11/KCTD21 are completely unrelated and present different biological partnerships, these mixed heteroassociations by means of the POZ/BTB domain are potentially able to expand the partnership of these proteins with the possibility to create new biological routes. The capability of the POZ/BTB domain of KCTDs to fold and unfold reversibly makes the process of subunit exchange between homomeric and heteromeric assemblies easier. Indeed, given the tight association of the subunits in the tetrameric/pentameric oligomers formed by the POZ/BTB domain, these replacements are likely associated with (partial) unfolding of the involved structural subunit. The ability of these domains to refold favors the formation of a combinatorial diversity of stable complexes whose composition depends on the local concentration of the different POZ/BTB domain and on their mutual affinity.

It is intriguing to speculate that the combination observed in KCTDs of well-conserved POZ/BTB, that assures the possibility to form heteromeric associations, coupled with diversified domains may be a more general and widespread feature among oligomeric proteins that allow intricate regulations of biological processes.

## Supplementary Material

The secondary structure prediction of KCTD6, an overlay of KCTD5BTB and KCTD6BTB CD spectra, the sequence alignment of KCTD6BTB and KCTD5BTB and a table reporting the comparison between theoretical molecular weights of KCTDs BTB/POZ assemblies and those calculated by light scattering and gel filtration analyses.

## Abbreviations

CD: Circular dichroism
KCTD5_BTB_: POZ/BTB domain of KCTD5
KCTD6_BTB_: POZ/BTB domain of KCTD6
GuHCl: Guanidine hydrochloride.
