# Supplementary material for: Thermal and Chemical Stability of Two Homologous POZ/BTB Domains of KCTD Proteins Characterized by a Different Oligomeric Organization
Source: Biomed Res Int. 2013 Nov 6;2013:162674. doi: 10.1155/2013/162674 (PMC3838848; doi:10.1155/2013/162674)
Supplement: Supplementary file 1 — The secondary structure prediction of KCTD6, an overlay of KCTD5BTB and KCTD6BTB CD spectra, the sequence alignment of KCTD6BTB and KCTD5BTB and a table reporting the comparison between theoretical molecular weights of KCTDs BTB/POZ assemblies and those calculated by light scattering and gel filtration analyses. [file 162674.f1.pdf]

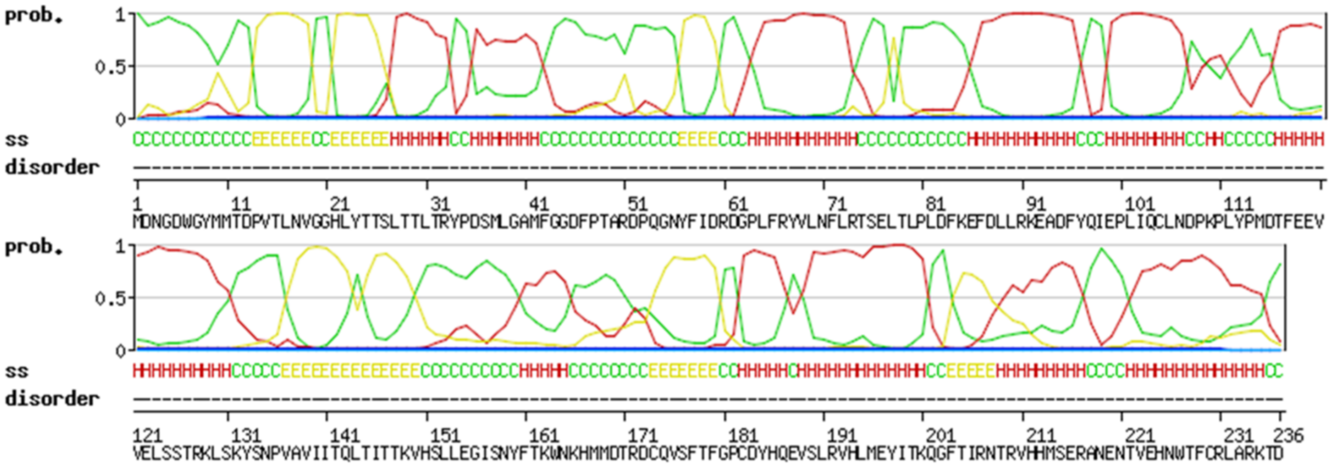

Fig. S1. Secondary structure prediction of KCTD6 (Swiss model server). In red (H) are indicated α-helix, in yellow (E) β-sheets and in green (C) coil regions.

```
KCTD5_BTB   40 SVSKWVRLNVGGTYFLTTRQTLCRDPKSFLYRLCQADPDLDSDKDETGAYLIDRDPTYFGPVLNY
                    : :::::  . :.  :: : : :.:  .  .:  . . .:  : :.::::   :  :::.
KCTD6_BTB   10   TDPVTLNVGGHLYTTSLTTLTRYPDSMLGAMFGGD--FPTARDPQGNYFIDRDGPLFRYVLNF


KCTD5_BTB        LRHGKLVINKDLAEEGVL-EEAEFYNITSLIKLVKDIRERDSKT     145
                 :: ..:..  :. :  .: .::.::.:  ::. ..:
KCTD6_BTB        LRTSELTLPLDFKEFDLLRKEADFYQIEPLIQCLND----KPLY     110
```

Fig. S2. Sequence alignment of KCTD6$_{BTB}$ and KCTD5$_{BTB}$. Residues involved in the interface of the pentameric KCTD5$_{BTB}$ are denoted in green. In red the Trp residue. The sequences reported for the two BTB/POZ domains correspond to the constructs expressed and characterized in this study.

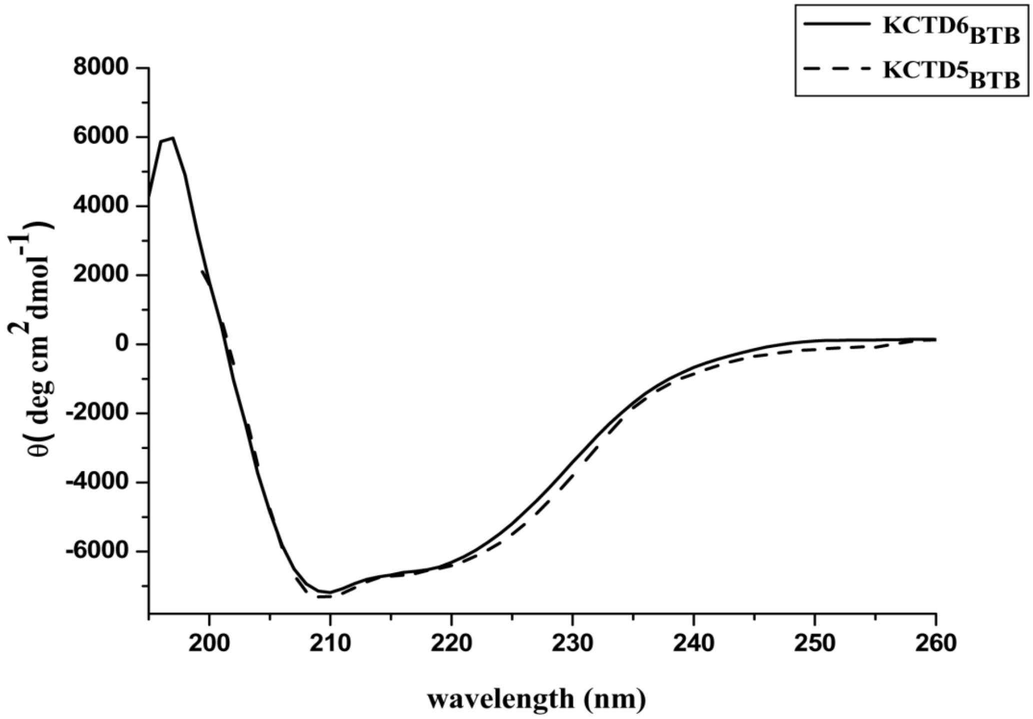

Fig S3. Overlay of $KCTD5_{BTB}$ and $KCTD6_{BTB}$ CD spectra, recorded at 20 °C.

Protein	Theoretical molecular weight (Da)	Calculated molecular weight (Da) by light scattering	Calculated molecular weight (Da) by gel filtration *
$KCTD5_{BTB}$	72140 (pentamer) – 57712 (tetramer)	73130 [6]	66400 [6]
$KCTD6_{BTB}$	59490 (pentamer) – 47592 (tetramer)	49990 [this work]	51100 [this work]
$KCTD11_{BTB}$	62120 (pentamer) – 49696 (tetramer)	50560 [6]	46900 [6]
$KCTD12_{BTB}$	63065 (pentamer) – 50452 (tetramer)	49320 [17]	44600 [Unpublished]

Table S1: Comparison between theoretical molecular weights of KCTDs BTB/POZ assemblies and those calculated by light scattering and gel filtration analyses. * The experimental data derived by gel filtration were calculated using the calibration formula y = -0.2952 x + 0.9006 [6].
